# Sex-related differences in patient characteristics, practice patterns and outcomes after bioprosthetic and mechanical aortic valve replacement

**DOI:** 10.1093/icvts/ivaf110

**Published:** 2025-05-08

**Authors:** Milos Matkovic, Igor Zivkovic, Slobodan Micovic, Ilija Bilbija, Petar Milacic, Nemanja Aleksic, Nemanja Milosevic, Milan Milojevic, Svetozar Putnik

**Affiliations:** Department of Cardiac Surgery, University Clinical Centre of Serbia, Belgrade, Serbia; Department of Cardiac Surgery, Institute for Cardiovascular Diseases Dedinje, Belgrade, Serbia; Department of Cardiac Surgery, Institute for Cardiovascular Diseases Dedinje, Belgrade, Serbia; Department of Cardiac Surgery, University Clinical Centre of Serbia, Belgrade, Serbia; Department of Cardiac Surgery, Institute for Cardiovascular Diseases Dedinje, Belgrade, Serbia; Department of Cardiac Surgery, University Clinical Centre of Serbia, Belgrade, Serbia; Department of Cardiac Surgery, Institute for Cardiovascular Diseases Dedinje, Belgrade, Serbia; Department of Cardiac Surgery, Institute for Cardiovascular Diseases Dedinje, Belgrade, Serbia; Department of Cardiovascular Research, Institute for Cardiovascular Diseases Dedinje, Belgrade, Serbia; Department of Cardiac Surgery, University Clinical Centre of Serbia, Belgrade, Serbia

**Keywords:** aortic valve replacement, sex difference, survival, mechanical prosthesis, biological prosthesis, patient–prosthesis mismatch

## Abstract

**OBJECTIVES:**

This study examines sex-based differences in baseline characteristics, practice patterns and mid-term survival following aortic valve replacement (AVR).

**METHODS:**

The study design included all consecutive patients from the AVR Registry with a 3-year follow-up. Patients were initially categorized by sex and prosthesis type. The primary end-point was 3-year all-cause mortality. Subgroup observations included the age-recommended threshold for mechanical prosthesis (<65 years) and the patient–prosthesis mismatch (PPM).

**RESULTS:**

The present study revealed that females (*n* = 517) compared to males (*n* = 732) were significantly older (67.2 ± 9.3 years vs 64.4 ± 12.2 years, *P* < 0.001), had higher body mass index (2.23 ± 7.2 cm^2^ vs 2.01 ± 0.2 cm^2^, *P* < 0.005) and lower left ventricular ejection fractions (51.8 ± 13.5% vs 57.7 ± 10.8%, *P* < 0.001) at the time of the index procedure. Additionally, females received significantly more bioprosthetic AVR than males (38.1% vs 32.6%, *P* = 0.040). There were no significant differences in 3-year mortality risk between males and females (14.6% vs 14.1%, *P* = 0.87). In subgroup analyses of patients with mechanical prostheses, females experienced a higher incidence of PPM than males (9.6% vs 2.2%, *P* < 0.001), whereas no significant difference was observed among those who received bioprosthetic valves. The male cohort observed reduced mortality associated with mechanical versus bioprostheses (hazard ratio [HR] 0.54, 95% confidence interval [CI] 0.36–0.81, *P* = 0.003). This effect was particularly significant in males under 65 (HR 0.27, 95% CI 0.11–0.68, *P* = 0.005). However, there was no difference in mortality related to prosthesis type in females, regardless of age group.

**CONCLUSIONS:**

At the time of referral for AVR, female patients were significantly older and had worse clinical profiles than male patients. Despite the higher rates of bioprosthetic valve implantation and PPM in females, mid-term survival rates were not significantly different. In contrast, males, especially those under 65, showed higher mortality following bioprosthetic AVR. These findings underscore the need for further research focusing on the sex-based treatment determinates in AVR.

## Graphical Abstract

**Figure:**
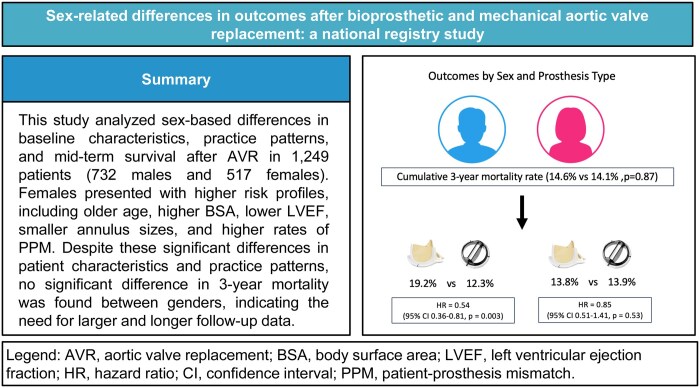

## INTRODUCTION

The total number of patients with aortic stenosis (AS) has steadily increased, reaching 9 million in 2019 and causing 120 000 deaths and 1.5 million disability-adjusted life years annually [1]. Aortic valve replacement (AVR) remains the gold standard of care for the majority of AS patients and is the second most frequent cardiac surgery procedure worldwide. Although clinical patterns and outcomes may differ between genders, prospective studies comparing gender-specific outcomes after AVR remain limited, as do pre-specified analyses from clinical trials. Several post hoc analyses of large clinical trials have revealed that females may be at a greater risk of adverse outcomes after AVR than males while also showing a lower risk of complications with transcatheter aortic valve implantation (TAVI) [2–4]. However, males are prevalent in multicentric clinical trials comparing TAVI with AVR, constituting up to 80% of the study population due to the higher prevalence of AS and more symptomatic disease, leading to easier recruitment [5]. This imbalance results in smaller sample sizes of females for analysing potential sex-related differences, making subgroup findings more challenging for interpretation [2–4, 6, 7].

Compared to males, females with AS tend to have a smaller aortic annulus, present at an older age, have lower body surface area (BSA), higher frailty scores and more comorbidities [3, 8]. Additionally, females are often referred for surgery at the more advanced stage of the disease [9]. These anatomical and clinical differences between sexes may significantly impact cardiac remodelling and fibrosis in AS, leading to a greater both short- and long-term risk of adverse events after AVR for females. Moreover, prosthesis–patient mismatch (PPM), a common and modifiable risk factor known to be associated with more cardiac events and worse haemodynamic performance [10], is suggested to be predominant in females compared to males [11, 12]. Importantly, current European Society of Cardiology (ESC)/European Association for Cardio-Thoracic Surgery (EACTS) clinical practice guidelines acknowledge these sex-specific disparities as critical knowledge gaps, offering no sex-specific recommendations for prosthesis type or age-based cut-offs in valve selection, and have underscored the urgent need to address these gender issues in future guideline revisions [13]. Finally, recent European data indicate that females have a significant life expectancy of around 4 years longer than males, potentially leading to undertreatment of this large patient population [14].

This lack of robust data complicates the ability to make different clinical decisions, underscoring the need for more comprehensive, sex-inclusive clinical research. Understanding these differences is crucial for optimizing treatment strategies and improving outcomes for males and females with AS. This study observes baseline differences, practice patterns, and mid-term survival differences by sex within the AVR cohort, offering insights to inform local practitioners and provide a hypothesis-generating foundation for future research efforts.

## METHODS

### Ethics statement

This study was conducted in accordance with the Declaration of Helsinki. Approval to use anonymized data without patient consent was granted by the Institutional Review Board of the University Clinical Centre of Serbia (IRB number KH1225/2023).

### Study design

This observational study uses data from a prospectively established multicentric cohort. All consecutive patients who underwent first-time AVR due to severe AS were screened between January 2015 and January 2020 (*N* = 2125). Patients with combined surgery or aortic insufficiency as the underlying pathology and redo cases were excluded. Additionally, those who missed the first hospital follow-up were not included in the cohort unless they were confirmed to be dead through the mortality registry. This resulted in 1249 patients with isolated AVR being included in the Registry. All patients with isolated AVR at 3-year follow-up were analysed according to their sex and the type of implanted prosthesis to derive prespecified subgroup analyses.

### Definitions and study end-points

This study’s primary outcome was all-cause mortality at 3 years. The prespecified subgroup analyses included: (1) the age-defined threshold for a mechanical prosthesis as recommended by the 2021 EACTS and the ESC Guidelines for the management of valvular heart disease (<65 years of age), and (2) the presence of PPM defined using the recently released formulas from the Valve Labelling Consortium [15].

The data were extracted from our National AVR Registry, a clinical registry that includes all patients undergoing AVR or repair at our high-volume institutions. Follow-up data were meticulously collected through clinical visits, telephone interviews and national mortality registries, ensuring no missing data regarding survival status. Preoperative characteristics were defined using standardized definitions, and the mortality risk was assessed using the EuroSCORE II [16]. All clinically gathered data were systematically registered in accordance with standardized national protocols.

### Patient–prosthesis mismatch

PPM was defined as an indexed effective orifice area (iEOA) < 0.85 cm^2^/m^2^. An iEOA between 0.85 cm^2^/m^2^ and 0.65 cm^2^/m^2^ was considered moderate PPM, while an iEOA < 0.65 cm^2^/m^2^ was regarded as severe PPM. PPM calculation was performed using the EOA value provided by the manufacturer for each prosthesis, divided by the patient’s BSA. Patients with mechanical and biological prostheses were compared according to the presence (iEOA < 0.85 cm^2^/m^2^) or absence (iEOA > 0.85 cm^2^/m^2^) of PPM.

### Surgical procedures

The surgical approaches used included standard median sternotomy and minimally invasive techniques, such as mini-sternotomy and mini-thoracotomy, which were used in 32.5% of cases. The choice of approach was at the discretion of the operating surgeon. According to local practice, mini-sternotomy was typically performed in a ‘J’ fashion at the fourth intercostal space, while mini-thoracotomy was conducted at the second or third intercostal space. A variety of prosthetic valves were used in the procedures. The mechanical prostheses included St Jude Regent, St Jude Masters (Abbott, Chicago, IL), ATS Open Pivot (Medtronic, Minneapolis, MN), On-X (CryoLife Inc., Kennesaw, GA) and Carbomedics (LivaNova, London, UK). The biological prostheses used were Hancock (Medtronic, Minneapolis, MN), Epic, Trifecta (Abbott, Chicago, IL), Crown PRT (LivaNova, London, UK), SoloSmart (LivaNova, London, UK) and the sutureless Perceval S valve (LivaNova, London, UK).

### Statistical analyses

Descriptive statistics were calculated for baseline demographic and clinical features as well as treatment outcomes. The normality of distribution was assessed using graphical methods, including histograms and Q-Q plots, and mathematical methods, specifically the Shapiro–Wilk test. Continuous variables were presented as means with standard deviations for normally distributed data or medians with 25th–75th percentiles for non-normally distributed data. Categorical variables were presented as numbers and percentages. Differences between groups were analysed using the Student’s t-test for continuous variables, the Mann–Whitney *U*-test when appropriate and the Pearson chi-squared test for categorical variables. No outcome comparisons beyond in-hospital and 3-year overall mortality were conducted between males and females due to marked differences in baseline characteristics, clinical presentation and treatment patterns in AVR. Therefore, the focus of the analysis remained within male and female subgroups to generate more accurate insights. Survival analysis was conducted using the Kaplan–Meier method, with comparisons between groups using the log-rank test. Hazard ratios (HRs) and corresponding 95% confidence intervals (CI) for the primary end-point and the secondary end-points were calculated relative to using the Cox proportional hazards model. The Schoenfeld residuals did not indicate any significant time-dependent effect. Post hoc selected patient characteristics with *P* < 0.15 in the univariate analysis were included in the multivariate logistic regression model to identify predictors of procedure outcomes, with gender forced into the model. The significance level was set at 0.05, and all tests were 2-sided. Statistical analyses were performed using IBM SPSS Statistics for Windows, version 21.0 (Armonk, NY, USA).

## RESULTS

### Baseline characteristics

Our study comprised 732 male and 517 female patients (59% vs 41%). Female patients were older (67.2 ± 9.3 vs 64.4 ± 12.2 years, *P* < 0.001), had higher body mass index (28.5 ± 13.5 vs 27.5 ± 5.1, *P* = 0.012), higher BSA (2.23 ± 7.2 vs 2.01 ± 0.2, *P* = 0.005), higher preoperative risk score (EuroSCORE II 1.85 ± 1.5 vs 1.78 ± 1.9, *P* = 0.005) and a lower left ventricular ejection fraction on presentation to AVR (51.8 ± 13.5 vs 57.7 ± 10.8, *P* < 0.001) (Table 1). Additionally, women had significantly smaller annulus size than the male study population (19.1 ± 2.3 mm vs 22.2 ± 3.4 mm, *P* < 0.001). The two groups did not differ in other baseline and procedural characteristics, including cardiopulmonary bypass time, aortic cross-clamp time or the intensive care length of stay (Table 1).

**Table 1:** Baseline and operative characteristics of male and female patients

	Male (*n* = 732)	Female (*n* = 517)	*P*-value
Age, years	64.4 + 12.2	67.2 + 9.3	0.001
Body surface area, m^2^	2.01 + 0.2	2.23 + 7.2	0.005
Body mass index	27.5 + 5.1	28.5 + 13.5	0.012
LVEF, %	57.7 + 10.8	51.8 + 13.5	0.001
EuroSCORE II	1.78 + 1.9	1.85 + 1.5	0.005
Hypertension	573 (78.2)	404 (78.1)	0.95
Hyperlipidaemia	272 (37.1)	218 (42.2)	0.14
COPD	72 (9.8)	46 (8.9)	0.59
CKD	70 (9.5)	40 (7.7)	0.20
Diabetes	127 (17.3)	115 (22.2)	0.03
PVD	39 (5.3)	31 (5.9)	0.60
Previous CVA	34 (4.6)	35 (6.7)	0.10
CPB time, min	90.3 + 31.4	89.7 + 27.3	0.86
Aortic cross-clamp time, min	65.3 + 22.6	61.6 + 23.1	0.65
Postoperative CVA	22 (3.1)	18 (3.4)	0.35
Intensive care time, days	2.8 + 2.6	2.6 + 2.4	0.39
Permanent pacemaker implantation	48 (6.5)	31 (5.9)	0.43
Hospital stay, days	9.4 + 6.8	9.2 + 5.3	0.24

Values are presented as *n* (%) or mean ± SD, as appropriate. CKD: chronic kidney disease; COPD: chronic obstructive pulmonary disease; CVA: cerebrovascular accident; CPB: cardiopulmonary bypass; DM: diabetes mellitus; HLP: hyperlipidaemia; HTA: arterial hypertension; EuroSCORE II: European System for Cardiac Operative Risk Evaluation II; ICU: intensive care unit; SD: standard deviation.

### Prosthesis types

#### Mechanical prosthesis

Mechanical prostheses were implanted in 67.4% of males and 61.9% of females (*P* = 0.19). The most frequently implanted prostheses were the St Jude Regent (Abbott, Chicago, IL) and St Jude Masters (Abbott, Chicago, IL), equally distributed between both groups. The ATS OpenPivot (Medtronic, Minneapolis, MN) was more frequently implanted in male patients (22.6% vs 9.3%, *P* < 0.001). The mean size of the implanted mechanical prosthesis was significantly smaller in females than in males (20.1 ± 1.6 mm vs 22.4 ± 1.9 mm, *P* < 0.001). In addition, PPM was more frequent among female patients than male patients who had mechanical prostheses implanted (9.6% vs 2.2%, *P* < 0.001) (Table 2).

**Table 2:** Sex-based distribution of prostheses by manufacturer and model and PPM rates

	Male (*n* = 732)	Female (*n* = 517)	*P*-value
Mechanical prosthesis	493 (67.4)	320 (61.9)	0.19
PPM	11 (2.2)	31 (9.6)	<0.001
St. Jude Masters (Abbott, Chicago, IL)	164 (33.4)	127 (39.7)	0.38
St. Jude Regent (Abbott, Chicago, IL)	211 (42.8)	158 (49.5)	0.47
ATS OpenPivot (Medtronic, Minneapolis, MN)	111 (22.6)	29 (9.3)	<0.001
ON-X (CryoLife Inc., Kennesaw, GA)	2 (0.5)	3 (1.0)	0.39
Carbomedics (LivaNova, London, UK)	3 (0.7)	1 (0.5)	0.50
Biological prosthesis	239 (32.6)	197 (38.1)	0.040
PPM	141 (58.9)	130 (65.9)	0.13
Hancock II (Medtronic, Minneapolis, MN)	118 (49.3)	51 (25.9)	<0.001
Epic (Abbott, Chicago, IL)	33 (13.9)	31 (15.9)	0.24
Trifecta (Abbott, Chicago, IL)	41 (17.2)	38 (19.4)	0.21
Crown PRT (LivaNova, London, UK)	9 (3.5)	11 (5.7)	0.26
SoloSmart (LivaNova, London, UK)	1 (0.6)	1 (0.5)	0.39
Perceval S (LivaNova, London, UK)	32 (13.5)	64 (32.6)	<0.001

Values are presented as n/N (%), as appropriate. PPM: patient–prosthesis mismatch.

#### Biological prosthesis

Biological prostheses were more frequently implanted in female than male patients (38.1% vs 32.6%, *P* = 0.040) (Table 2). The frequency of PPM did not vary significantly between male and female groups (58.9% vs 65.9%, *P* = 0.13). The most frequently implanted biological prosthesis type was Hancock II (Medtronic, Minneapolis, MN), which was almost double more frequently implanted in males than in females (49.3% vs 25.9%, *P* < 0.001). Other prosthesis types had a non-statistically significant distribution between the groups, except for the Perceval S sutureless valve, which was significantly more frequently implanted in female patients than male patients (32.6% vs 13.5%, *P* < 0.001). Regarding the size of the Perceval valve, the most frequently implanted size in females was size S (45.3% vs 6.5%, *P* < 0.001), while in males, it was size L (46.8% vs 20%, *P* < 0.001). The mean size of other biological prostheses implanted was significantly smaller in females compared to males (19.8 ± 3.1 mm vs 21.4 ± 5.3 mm, *P* < 0.001).

### Mortality differences by sex and type of prosthesis

**Figure 1:**
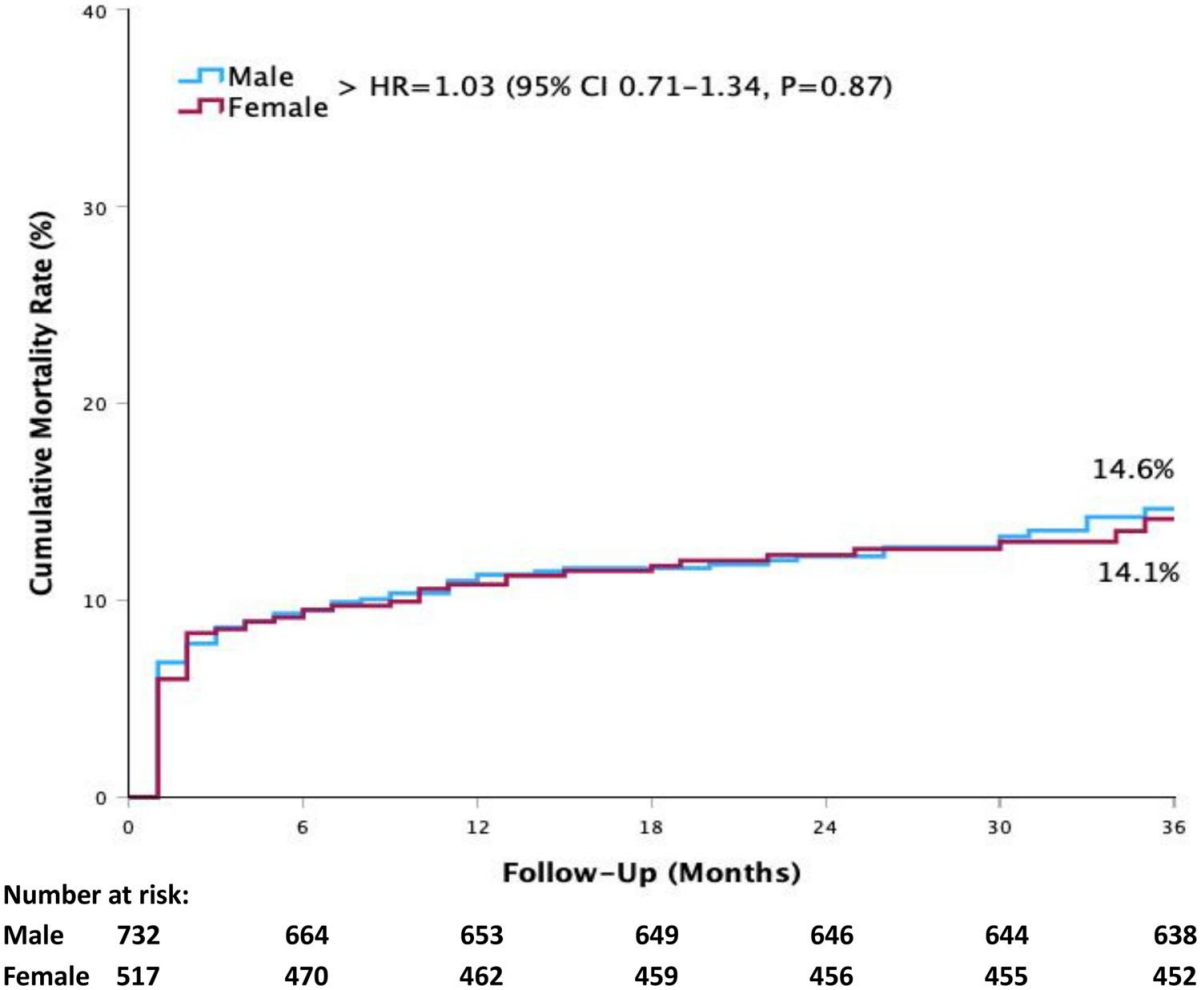
Kaplan–Meier cumulative mortality rates according to sex of patients undergoing surgical aortic valve replacement. HR: hazard ratio

Vital status at 3 years was obtained for all patients, ensuring complete follow-up for the selected cohort. The analysis of in-hospital mortality did not show a significant difference between male and female patients (1.4% vs 1.8%, *P* = 0.53). There were no statistically significant differences up to 3 years (*P* = 0.87), with 3-year mortality estimates of 14.6% and 14.1% (Fig. 1). Moreover, multivariable logistic regression did not reveal gender as an independent predictor of 3-year mortality (HR = 0.75, 95% CI 0.51–1.10, *P* = 0.14) (Table 3).

**Table 3:** Univariate and multivariate analysis of predictors for procedural success

Variables	Univariate analysis	Multivariate analysis
	HR (95% CI)	HR (95% CI, [beta coefficient])
Age, years	1.04 (1.02–1.06, P < 0.001)	1.04 (1.02–1.06, *P* < 0.001 [0.039])
LVEF, %	0.99 (0.98–1.00, *P* = 0.077)	1.00 (0.98–1.02, *P* = 0.94 [0.001])
EuroSCORE II	1.11 (1.06–1.17, *P* < 0.001)	1.13 (1.05–1.20, *P* < 0.001 [0.119])
Hypertension	1.45 (0.95–2.20, *P* = 0.085)	1.38 (0.84–2.28, *P* = 0.20 [0.324])
COPD	2.05 (1.01–4.17, *P* = 0.048)	2.00 (0.97–4.11, *P* = 0.060 [0.693])
CKD	1.79 (1.15–2.78, *P* = 0.010)	1.66 (1.02–2.71, *P* = 0.040 [0.509])
Bioprosthetic valve	1.54 (1.12–2.12, *P* = 0.007)	1.25 (0.84–1.86, *P* = 0.26 [0.225])
Male vs female	0.97 (0.71–1.34, *P* = 0.87)	0.75 (0.51–1.10, *P* = 0.14 [−0.287])

CI: confidence interval; COPD: chronic obstructive pulmonary disease; CKD: chronic kidney disease; LVEF: left ventricular ejection fraction; HR: hazard ratio.

**Figure 2:**
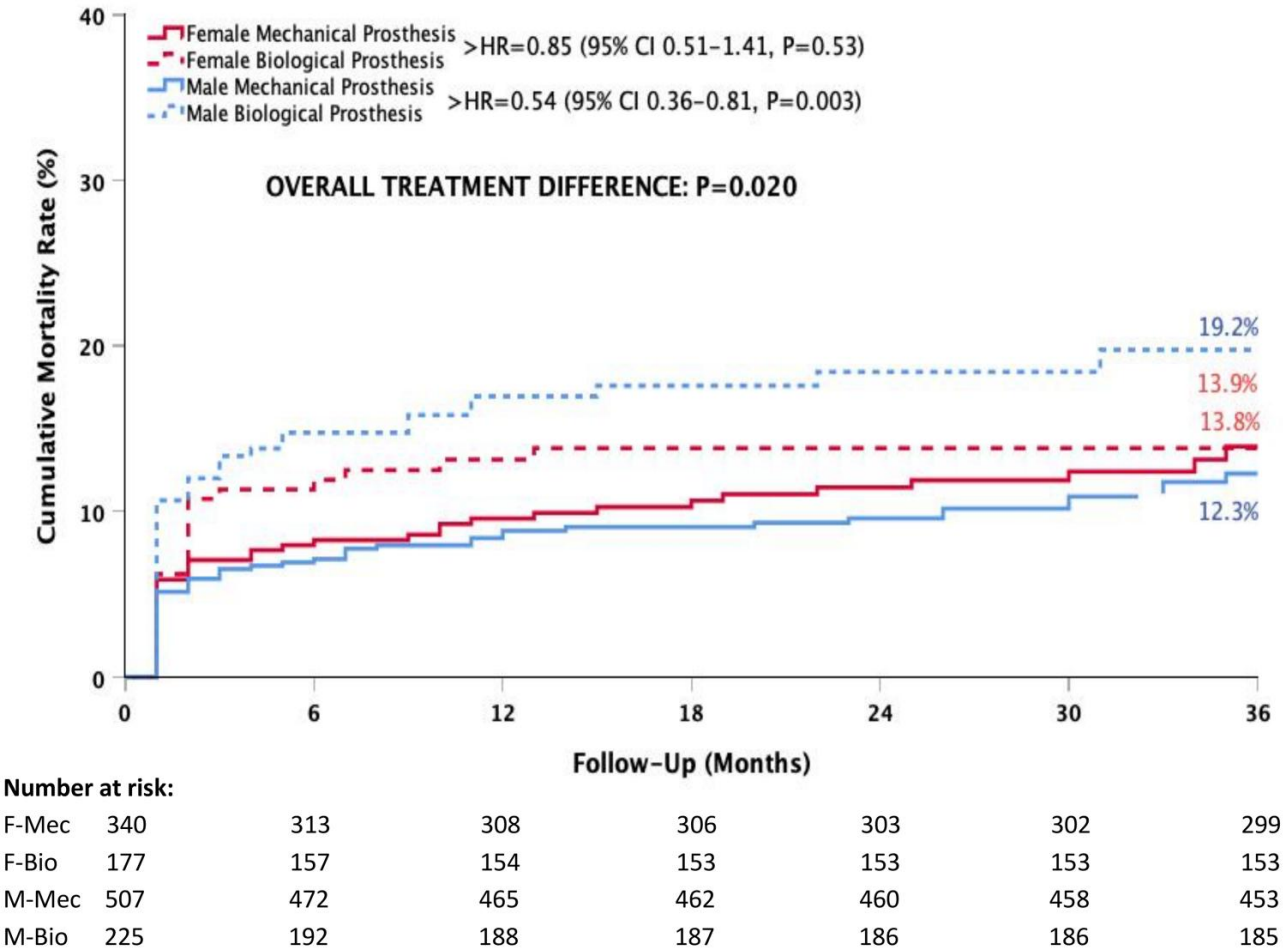
Kaplan–Meier cumulative mortality rates according to sex and prosthesis type in patients undergoing surgical aortic valve replacement. HR: hazard ratio

Among male patients, those who received biological prostheses had a higher mortality rate than those who received mechanical prostheses (19.2% vs 12.3%, HR = 0.54, 95% CI 0.36–0.81, *P* = 0.003) (Fig. 2). Male patients with mechanical valves were significantly younger than those with biological valves (60.1 ± 12.9 years vs 71.4 ± 7.6 years, *P* < 0.001). In the female group, there was no statistical difference in mortality between biological and mechanical prostheses (13.9% vs 13.8%, HR = 0.85, 95% CI 0.51–1.41, *P* = 0.53) (Fig. 2). However, female patients with mechanical prosthesis were significantly younger than those with biological prosthesis (65.1 ± 10.4 years vs 71.7 ± 5.7 years, *P* < 0.001).

#### Subgroup analyses

**Figure 3:**
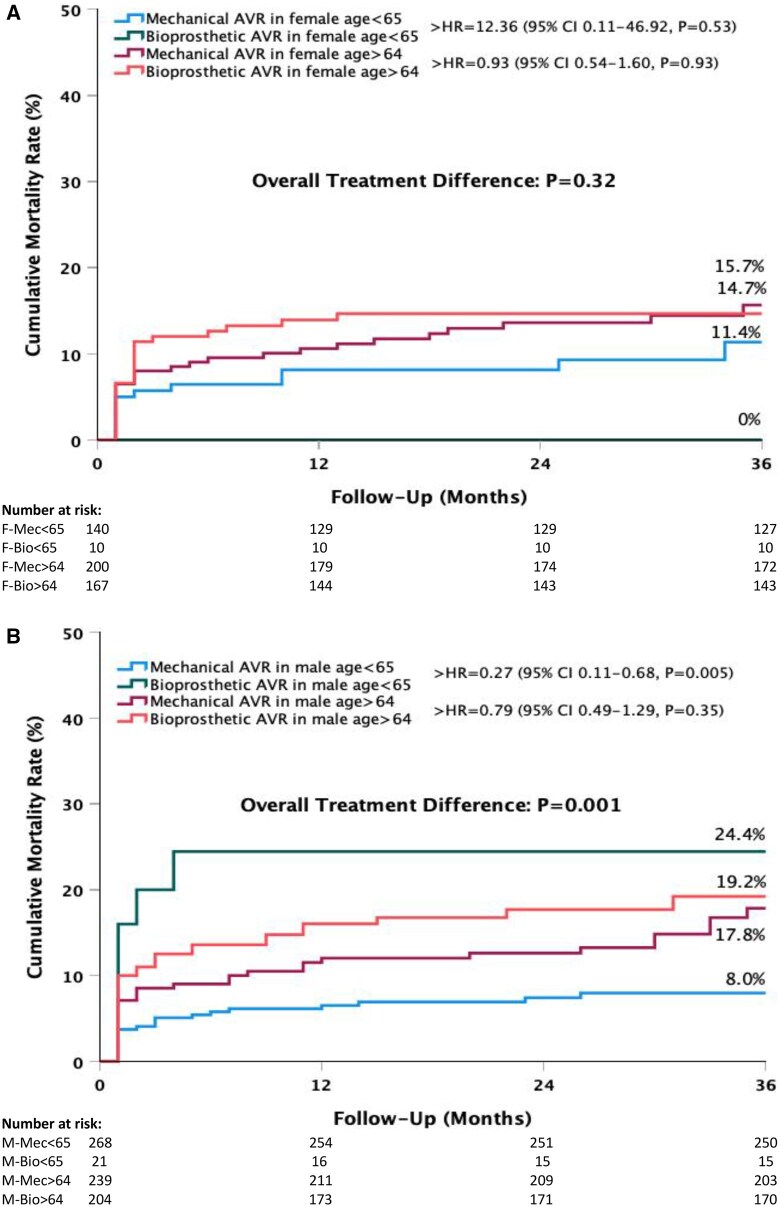
Kaplan–Meier cumulative mortality rates by the prosthesis type and 65-year age cut-off in the females (**A**) and males (**B**). AVR: aortic valve replacement; HR: hazard ratio

When the 65-year age cut-off was applied in the female patient group, there was no statistically significant difference in mortality rates between those who received bioprosthetic and mechanical valves (Fig. 3A). In the male group under 65 years of age, a higher mortality rate was observed in patients who received biological prostheses compared to those who received mechanical prostheses (HR = 0.27, 95% CI 0.11–0.68, *P* = 0.005). In males over 65, there was no statistically significant difference in mortality rates regardless of the type of prosthesis implanted, biological vs mechanical (HR = 0.79, 95% CI 0.49–1.29, *P* = 0.35) (Fig. 3B).

**Figure 4:**
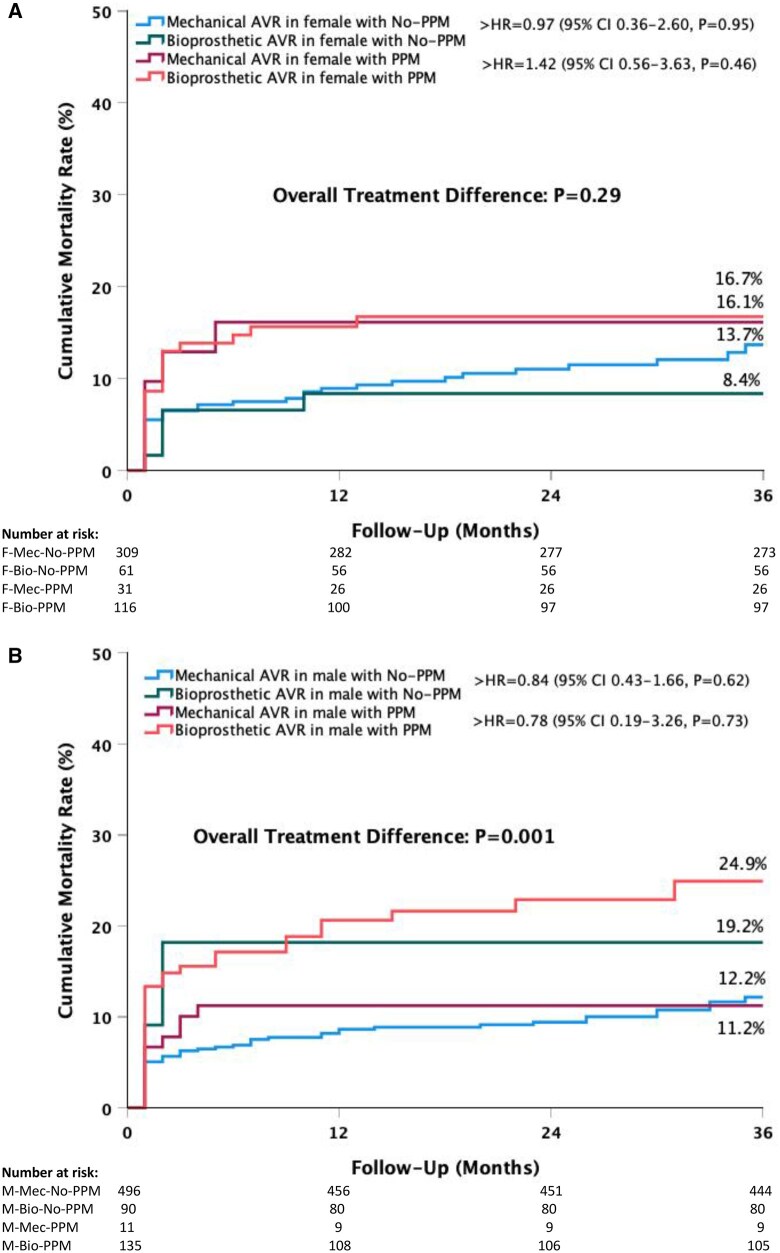
Kaplan–Meier cumulative mortality rates by the prosthesis type and presence of patient-prosthesis mismatch in the females (**A**) and males (**B**). AVR: aortic valve replacement; HR: hazard ratio

There were no significant differences in 3-year mortality risk in the presence of PPM after mechanical or biological prostheses implantation in both females (Fig. 4A) and males (Fig. 4B).

Analysing mid-term mortality rates by surgical approach revealed no significant differences between groups. For males, mortality was 13.7% in the full sternotomy group and 14.8% in the minimally invasive group, which includes mini-sternotomy and right anterior thoracotomy (HR = 0.68, 95% CI 0.42–1.05, *P* = 0.42). For females, the rates were 13.9% for full sternotomy and 15.1% for minimally invasive procedures (HR = 0.75, 95% CI 0.52–1.14, *P* = 0.54).

## DISCUSSION

This research explored sex-based differences in baseline characteristics, practice patterns and mid-term survival associated with AVR. The key findings include: (i) female patients were older and had worse clinical risk profiles at the time of index AVR procedure; (ii) there was a significantly higher implantation rate of biological prostheses in female than male patients; (iii) no significant difference was observed between males and females in 3-year mortality risks; (iv) males who underwent bioprosthetic AVR had a significantly higher mortality risk compared to those who received mechanical valve prostheses, while in females, there was no difference in survival according to the type of prosthesis; (vi) the mortality difference favouring mechanical prostheses was particularly pronounced in patients aged < 65 and (vii) the incidence of PPM was higher in females than in males; however, this did not result in a significant mid-term survival disadvantage, regardless of prosthesis type.

Several registry-based analyses reveal that females are less often treated with AVR compared to males, aligning with the demographic distribution observed in our study population [17–19]. Chaker *et al.* analysed 166 809 AVR patients from 2003 to 2014 in the USA, finding a male-to-female ratio of 63% to 37% [17]. Our results mirror these findings, showing that females were older and had higher baseline operative risks. This disparity in clinical profiles, marked by worse risk scores in females, may stem from their typically later referral during the disease course, which is characterized by more advanced haemodynamic compromise and a higher incidence of cardiac insufficiency and decompensation [20]. These later referrals could relate to the distinct pathophysiology of AS in females, influenced by hormonal factors that typically delay the onset of heart disease until later in life. Additionally, females with AS often present with atypical symptoms and, due to a smaller aortic root and left ventricular cavity, which results in a reduced stroke volume, they are frequently misdiagnosed with less severe AS. This diagnostic challenge is further compounded by the absence of gender-specific cut-off values for stroke volume [21, 22]. Furthermore, females tend to show more leaflet calcification and fibrosis than males for the same EOA size. This observation persists even after adjusting for a smaller BSA, which may be another result of their later referral in the more advanced stage of the disease [23].

Building on the observation that females exhibit more advanced leaflet calcification and fibrosis, it is important to note the anatomical differences predisposing female patients to a higher risk of PPM [6]. Due to a smaller annulus and lower BSA, females often face increased challenges with prosthetic valve fitting. In the present study, despite a higher BSA in female patients, the annulus size was significantly smaller than in male patients. This could explain the higher PPM rate, even in the mechanical prosthesis group. Bonderman *et al.* reported a higher incidence of PPM (58% with PPM in women versus 36% in men, *P* < 0.001) and bioprosthetic valve implantations in females (82% in females versus 62% in males, *P* < 0.001), which they attributed to the smaller aortic root sizes typical among this group [11]. Our study corroborates these findings, noting a higher incidence of PPM in female patients than males, especially with mechanical valves. Interestingly, despite these disparities, long-term mortality rates do not seem to be significantly impacted by PPM across genders. Tully *et al.* found no significant difference in long-term mortality between males and females with PPM [24]. Our research similarly indicates no significant 3-year mortality differences in male and female patients based on PPM and the type of prosthesis used. Although studies vary in their conclusions about the impact of PPM on clinical outcomes, there is a general agreement that PPM should be avoided to prevent the complications associated with increased prosthetic gradients following AVR [25, 26]. The relatively higher PPM rates in this study likely contributed to the longer average length of stay, influenced by the national policy at the time, which required ICU stays of 2 days and discharged on day 7, establishing a minimum stay. Adverse events, including PPM implantation, extended this to an average of 9 days. Today, advancements like remote ECG monitoring have enabled earlier discharges, often on day 5 or even day 4. Nevertheless, it is important to investigate the impact of PPM over a longer follow-up period, as a recent study from the SWEDEHEART registry found no significant effect of PPM on survival after 10 years [27]. Thus, a 3-year follow-up represents an important limitation when analysing the impact of PPM on survival after AVR.

When considering survival differences after AVR, it is imperative to consider the interplay of age and prosthesis type; however, gender may also play a critical role in treatment decision-making. Çelik *et al.* analysed a Rotterdam cohort of 3462 AVR patients over a follow-up period extending to 30 years. This study found that female patients were typically older at the time of surgery. Despite their older age and after adjustments for age and gender, these female patients demonstrated significantly better 20-year survival rates than males [20]. Conversely, Chaker *et al.* reported a 30% lower in-hospital survival relative measure rate for female patients compared to male patients after AVR, a disparity that persists at the 1-year follow-up, even after adjustments for age [17]. Our analysis revealed no significant differences in overall 3-year survival between males and females, consistent with the 2-year findings reported after merging the INSPIRIS RESILIA Durability Registry (INDURE) and the IMPACT registries [19]. While survival rates did not differ among female patients based on the type of prosthesis used, male patients with bioprostheses exhibited lower survival rates than those with mechanical valves. When a 65-year age cut-off was applied, we observed no survival differences in patients over 65, nor were there any overall treatment disparities. However, in males under 65, those with bioprostheses had significantly lower survival than those with mechanical valves, while in the same female cohort under 65, survival rates did not vary with valve type. These findings emphasize the necessity of considering life expectancy and survival rates when selecting prostheses for different patient groups, highlighting the importance of gender in these clinical decisions.

It should also be noted that our results may have been influenced by the types of bioprostheses used, as there was a high usage rate of Hancock II and Trifecta valves in our study population. This is particularly relevant in the case of Trifecta implantation, as recent data suggest superior outcomes with other types of bioprostheses, especially Resilia and PERIMOUNT Magna Ease valves [28].

Given these observed treatment differences, which may stem from various pathophysiological factors and patient presentations, and the exponential growth of TAVI procedures despite limited data, further studies are urgently needed. Such research is essential to distinguish the best treatment options for specific patient groups, including minorities, ensuring optimal outcomes.

### Study limitations

As this is an observational study, the findings should be interpreted cautiously and several important limitations must be noted. First, the results should be interpreted as hypothesis-generating due to the exploratory nature of the data analysis. This suggests that the findings are preliminary and intended to guide quality improvement of local practices and future studies rather than to provide definitive conclusions. Second, due to limited resources and the national mortality reporting system constraints, we could not determine specific causes of death and other important outcome measures, including hospital readmission. This limitation reduces the granularity of our mortality data and our ability to establish causality. Third, substantial variability in perioperative procedures likely contributed to some of the observed cerebrovascular accident (CVA) rates. For instance, transoesophageal echocardiography was not standardized during the study period, although it was utilized in many cases. Similarly, CO_2_ insufflation was implemented selectively, depending on the preferences of individual surgeons, and the routine use of computed tomography or other standardized preventive measures for CVA reduction was not consistently adopted. These variations, combined with contributions from over 50 practising surgeons, underscore the heterogeneity of practice patterns and their potential impact on outcomes, including the higher observed CVA rate. Fourth, inherent biases and possible confounding factors within registry data may have influenced our findings. Despite the potential for biases, the decision to include only patients with at least 3 years of follow-up was influenced by multiple considerations to ensure our findings’ robustness and validity. Primarily, our study’s objective was to examine baseline and practice pattern differences but also to accurately assess mid-term survival outcomes, where longer-term data are essential to minimize bias in survival analysis. This approach helps prevent the potential misclassification of survival status that could occur with incomplete data, thus preserving the scientific integrity of the analysis. Moreover, multivariable or propensity-matched adjustments were not performed due to the lack of preselection of variables and the significant heterogeneity among the limited number of participants, as this could have introduced additional biases or masked meaningful interactions, thereby representing a significant limitation of the present study. Additionally, longer follow-up is needed to assess the impact of PPM on survival after AVR more accurately. Fourth, another important limitation is the diverse types of bioprostheses used in the study, as the high implantation rates of Trifecta and Hancock II prostheses might influence the outcomes. Additionally, sutureless valves were considerably used during this period, per the discretion of the operating surgeon, further impacting the interpretation of the findings [29, 30]. Moreover, several newer generation bioprosthetic valves known to be associated with improved durability and haemodynamics, such as Magna Ease and Resilia valves, were unavailable in our national market, which could have further impacted the generalizability of the results [31–33]. Finally, more extensive research is needed to validate these findings before they can be applied clinically in other populations.

## CONCLUSION

The present observational study revealed that female patients undergoing AVR were typically referred later and presented with more advanced clinical risk profiles at the time of index hospitalization compared to male patients. This scenario may often result in a higher rate of bioprosthesis implantation and PPM among females. Despite these initial differences, there was no statistically significant disparity in 3-year mortality risks between genders. However, among male patients under 65, those who received bioprosthetic AVR encountered a significantly higher mortality risk compared to those with mechanical valve prostheses, consistent with ESC/EACTS guidelines recommending mechanical valves for this population. The observed sex differences in prevalence, patient characteristics, clinical presentation and even mid-term outcomes in AVR highlight the critical need for tailored patient-centred and individualized treatment strategies. These findings suggest the importance of customizing care approaches and reinforce the need for further prospective studies and international data-sharing efforts to understand and address sex-related differences in patients requiring AVR entirely.

## Data Availability

No data were produced for the content of this paper.

## ABBREVIATIONS

AS: Aortic stenosis
AVR: Aortic valve replacement
BSA: Body surface area
CI: Confidence interval
CVA: Cerebrovascular accident
EACTS: European Association for Cardio-Thoracic Surgery
EOA: Effective orifice area
ESC: European Society of Cardiology
HR: Hazard ratio
iEOA: Indexed effective orifice area
TAVI: Transcatheter aortic valve implantation
